# Genome-wide association study reveals novel QTLs and candidate genes for seed vigor in rice

**DOI:** 10.3389/fpls.2022.1005203

**Published:** 2022-10-26

**Authors:** Liping Dai, Xueli Lu, Lan Shen, Longbiao Guo, Guangheng Zhang, Zhenyu Gao, Li Zhu, Jiang Hu, Guojun Dong, Deyong Ren, Qiang Zhang, Dali Zeng, Qian Qian, Qing Li

**Affiliations:** ^1^ State Key Laboratory for Rice Biology, China National Rice Research Institute, Chinese Academy of Agricultural Sciences, Hangzhou, China; ^2^ The Key Laboratory for Quality Improvement of Agricultural Products of Zhejiang Province, College of Advanced Agricultural Sciences, Zhejiang A & F University, Hangzhou, China

**Keywords:** rice, GWAS, seed vigor, direct seeding, haplotype

## Abstract

Highly seed vigor (SV) is essential for rice direct seeding (DS). Understanding the genetic mechanism of SV-related traits could contribute to increasing the efficiency of DS. However, only a few genes responsible for SV have been determined in rice, and the regulatory network of SV remains obscure. In this study, the seed germination rate (GR), seedling shoot length (SL), and shoot fresh weight (FW) related to SV traits were measured, and a genome-wide association study (GWAS) was conducted to detect high-quality loci responsible for SV using a panel of 346 diverse accessions. A total of 51 significant SNPs were identified and arranged into six quantitative trait locus (QTL) regions, including one (*qGR1-1*), two (*qSL1-1*, *qSL1-2*), and three (*qFW1-1*, *qFW4-1*, and *qFW7-1*) QTLs associated with GR, SL, and FW respectively, which were further validated using chromosome segment substitution lines (CSSLs). Integrating gene expression, gene annotation, and haplotype analysis, we found 21 strong candidate genes significantly associated with SV. In addition, the SV-related functions of *LOC_Os01g11270* and *LOC_Os01g55240* were further verified by corresponding CRISPR/Cas9 gene-edited mutants. Thus, these results provide clues for elucidating the genetic basis of SV control. The candidate genes or QTLs would be helpful for improving DS by molecular marker-assisted selection (MAS) breeding in rice.

## Introduction

Rice direct seeding (DS) technology, in which seeds are directly sown in the field without seedling raising or transplanting, provides an advanced and simplified cultivation technology for mechanized rice production. With the decrease in the rural labor force, the rise in labor cost, and the development of mechanized rice planting technology, rice planting in Asia, the main rice-producing area, has a trend of developing from traditional or mechanical transplanting to mechanical direct planting due to its labor- and cost-saving. However, DS requires the strong seed vigor (SV) of cultivated rice varieties, including high and uniform seed germination rate, strong seedling establishment and growth capacity, and enhanced stress tolerances. Most transplanting rice varieties on the market, including conventional rice, hybrid rice, and super rice, cannot be used for DS due to their poor SV. Thus, it is urgent to develop high SV varieties suitable for rice mechanized direct seeding through molecular-assisted selective breeding and biotechnology breeding, which depends on understanding the regulatory mechanism of SV-related traits and identifying the essential genes underlying these traits.

SV is a comprehensive trait affected by genetic and environmental factors during seed development and maturation. At present, a large number of QTLs for SV-related characteristics, such as seed germination rate (Wang et al., 2010; Jiang et al., 2020; Najeeb et al., 2020), coleoptile/seedling length (Dang et al., 2014; Xie et al., 2014; Jin et al., 2018), and seedling weight (Zhang et al., 2017; Najeeb et al., 2020), have been found in rice. Most of these QTLs were identified by bi-parental mapping with traditional markers. However, due to the low density of traditional markers, only a few QTLs or genes for SV have been fine-mapped. For instance, Fujino et al. isolated *qLTG3-1*, a QTL associated with low-temperature germinability. It may accelerate vacuolization and weaken the tissues covering the embryo during seed germination, thereby reducing the mechanical resistance to the growth potential of coleoptile (Fujino et al., 2008). In addition, *qSE3*, a major QTL for rice seed germination and seedling establishment under salinity stress, was identified as a K^+^ transporter OsHAK21 (He et al., 2019a). Physiological analysis revealed that *qSE3* improved seed germination and seedling establishment against salinity stress through mediating seed physiological states, including increasing K^+^ and Na^+^ uptake, promoting abscisic acid (ABA) biosynthesis, and reducing reactive oxygen species (ROS) accumulation. Very recently, *OsHIPL1*, which encodes a hedgehog-interacting protein-like 1 protein, was cloned as a causal gene for the major QTL *qSV3* associated with rice SV (He et al., 2022). And *OsHIPL1* may play its role by modulating ABA levels and *OsABIs* expression during rice seed germination.

Genome-wide association study (GWAS) has become an effective way to dissect complex traits and identify corresponding loci or candidate genes. Several QTLs or genes for SV have been detected *via* GWAS in rice. For example, four germination rate (GR)-related QTLs in rice were revealed by GWAS, and *OsOMT*, encoding the 2-oxoglutarate/malate translocator, was further validated as a causal gene for *qGR11*. *OsOMT* played an essential role in GR by modulating amino acid levels, and the processes of glycolysis and tricarboxylic acid cycle (Li et al., 2021a). Yang et al. identified 19 QTLs associated with rice seed germination by GWAS. They further found that *OsPK5* was a positive regulator of rice seed germination by encoding a pyruvate kinase and thereby modulating glycolytic metabolism and abscisic (ABA)/gibberellins (GA) balance (Yang et al., 2022). In addition, *OsCDP3.10*, encoding a cupin domain protein, was identified from *qSP3*, a significant QTL for rice seedling percentage by GWAS (Peng et al., 2022). Functional analysis revealed that *OsCDP3.10* affected rice SV mainly by modulating the levels of amino acids and H_2_O_2_ in the germinating seeds. These studies demonstrated that GWAS is an effective way to identify QTLs/genes for SV in rice.

However, so far, almost all studies on GWAS related to SV have been carried out in Petri dishes and under constant temperature conditions, which cannot fully simulate the natural environment of the field. Therefore, the present study intends to apply GWAS to identify QTLs/genes for rice SV in the field. We surveyed the GR, seedling shoot length (SL), and shoot fresh weight (FW) of 346 rice accessions in the field, and performed GWAS analysis to identify reliable loci responsible for rice SV. In total, six QTLs were detected, including one for GR, two for SL, and three for FW. Combining gene expression, gene annotation, and haplotype analysis, we screened 21 candidate genes significantly associated with SV from these QTLs, among which *LOC_Os01g11270* and *LOC_Os01g55240* were further validated by corresponding CRISPR/Cas9 gene-edited mutants. The detected QTLs and candidate genes will enhance our understanding of the genetic and molecular basis for SV and provide valuable resources for applying these potential elite loci in rice molecular breeding.

## Materials and methods

### Plant material and phenotyping

The 346 representative accessions distributed worldwide were selected for the GWAS panel, including 213 *indica* and 133 *japonica* rice accessions (**Supplementary Table 1**). All seeds of these accessions were harvested at their maturity stage in September and October 2020, dried, and stored in a refrigeration house. To break seed dormancy, these seeds were dried at 42 °C for seven days before sowing (He et al., 2019b; He et al., 2022). When seeds are sown, the waterlogged paddy field is silty and sticky without standing water. In May 2021, 50 well-filled seeds of each accession were evenly sown in a circular box (6.4 cm with a diameter) on the surface of the paddy field at China National Rice Research Institute (Hangzhou, Zhejiang, China), following a randomized complete block experimental design with three replications. One week after sowing, we began to slowly saturate the paddy field, maintaining a thin layer of water with a depth of 1-2 cm on the soil’s surface. After direct sowing for two weeks, the data of GR, SL, and FW were measured. The temperature during this period was 17 °C ~ 26 °C. The seed was regarded as germination when the coleoptile or radicle length was longer than 1 cm, and GR was defined as the ratio of the number of germinated seeds to the total number of seeds. Five plants for each accession were randomly selected to measure the SL and FW (shoot fresh weight of all five plants). The mean data of GR, SL, and FW were presented and obtained from the three replicates, including 344, 314, and 313 mean values for GR, SL, and FW, respectively (**Supplementary Table 1**). The lack of mean GR data for two accessions was due to the large SD of three replicates, which was considered inaccurate and consequently discarded. Similarly, the mean FW value of D188 accession, and SL and FW values of D299 accession were also discarded. In addition, to eliminate the influence of germination time on SL and FW, the SL and FW of 31 accessions with low GR (<38%) and shallow emergence rate one week after sowing were not recorded. These missing GR, SL, and FW data were marked as NA in **Supplementary Table 1**.

Chromosome segment substitution lines (CSSLs) used for *qGR1-1, qSL1-1, qSL1-2*, *qFW1-1*, *qFW4-1*, and *qFW7-1* validation were constructed by a combination of backcross and molecular maker-assisted selection (Dai et al., 2020). After backcrossing four times, each CSSL has a genetic background with recurrent receptor parent 9311 and only carries a target chromosomal segment from the donor parent Nipponbare (NPB). After drying at 42 °C for seven days, 50 well-filled seeds per replicate of 9311 and CSSLs were directly sown in ddH_2_O at 30 °C for four days, then transplanted in a conventional Kimura B hydroponic medium at 30 °C for six days (maintain culture in ddH_2_O for GR). The GR, SL, and FW data for each CSSL and its recurrent parent 9311 were collected ten days after cultivation. Three biological replicates were performed for GR and FW, and ten replicates were used for SL.

The CRISPR/Cas9-mediated gene editing mutants for *LOC_Os01g11270* and *LOC_Os01g55240* were obtained from Weimi Biotechnology Co., Ltd. (Lu et al., 2017). And the cultivation and measurement methods are the same as those of the CSSLs.

### Genome resequencing and SNP genotyping

The genomic DNA of each accession for the GWAS population was extracted by the cetyltrimethylammonium bromide (CTAB) method (Murray and Thompson, 1980). The resequencing library was constructed according to the manufacturer’s instructions (Illumina, San Diego, CA, USA). The paired-end reads in each library were generated by Illumina NovaSeq 6000 platform at Berry Genomics Company (http://www.berrygenomics.com/,Beijing,China). The clean reads were aligned to the NPB reference genome (IRGSP-1.0) with Burrows-Wheeler Aligner (BWA) (Li and Durbin, 2009). Genome Analysis Toolkit software (GATK) (Van der Auwera et al., 2013) was used for single nucleotide polymorphism (SNP) calling. The SNP annotations were analyzed by SnpEff (Cingolani et al., 2012) based on the GFF3 files of the NPB reference genome. A total of 2,748,212 high-quality SNPs with minor allele frequency (MAF) greater than 5% and a missing rate less than 20% were screened for further GWAS.

### Population genetics analysis

We randomly selected 100,000 SNPs with a MAF > 5% and a missing rate < 20% to build a phylogenetic tree using SNPhylo (Lee et al., 2014; Li et al., 2021b; Feng et al., 2022), and plotted it with the R package of ‘ggtree’ (Yu et al., 2017). The Principal component analysis (PCA) and population structure analysis for all accessions were calculated by Plink (Purcell et al., 2007) and ADMIXTURE (Alexander et al., 2009).

### Genome-wide association study

GWAS was conducted using vcf2gwas with a linear mixed model (Vogt et al., 2022). GR, SL, and FW values for 346 rice accessions were used as input data. Based on the Bonferroni-corrected method and previous reports (Yang et al., 2014; Li et al., 2021b; Feng et al., 2022), the *P-value* of the whole-genome significance cutoff was set as 1/n (n = the number of SNPs used in association analysis); that is, the threshold of -log_10_ (*P*) was about 6.4. Manhattan and quantile-quantile (Q-Q) plots were generated using the R package of Cmplot (https://github.com/YinLiLin/CMplot). Based on the previous report, the 200-kb upstream and downstream significant SNP site was regarded as a QTL region, and adjacent overlapped regions were merged into the same QTL (Li et al., 2022).

### Haplotype analysis

The SNPs in the regions of 2-kb upstream, CDS, and 1-kb downstream sequence of each gene were selected for haplotype analysis. The rare haplotype with a frequency lower than ten were discarded. The top two or three haplotypes with the highest frequency were chosen for further phenotypic association analysis.

## Result

### Phenotypic variation of SV-related traits in 346 rice accessions

**Figure 1:**
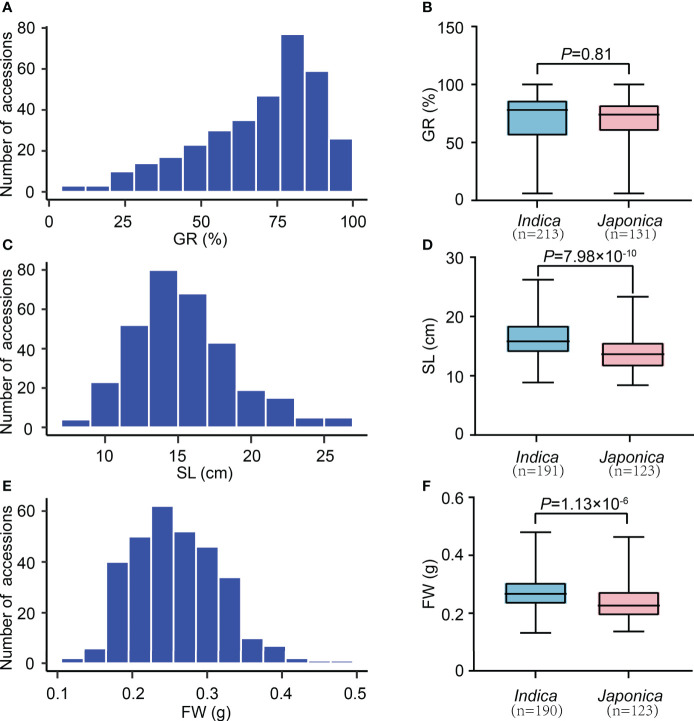
The variation of SV-related traits in 346 rice accessions. **(A)** Distribution of GR values. **(C)** Distribution of SL values. **(E)** Distribution of FW values. **(B)** Comparison of GR values between *indica* and *japonica*. **(D)** Comparison of SL values between *indica* and *japonica*. **(F)** Comparison of FW values between *indica* and *japonica.* n indicates the number of accessions. The p-value is obtained from the T-test.

We observed significant variations of three SV-related traits (GR, SL, and FW) among 346 rice accessions. In detail, the GR ranged from 6.00 to 100.00%, with an average of 69.74% and a variation coefficient of 28.69% (**Table 1**). The SL changed from 8.40 to 26.20 cm, with a mean value of 15.42 cm and a variation coefficient of 22.65%. The FW varied from 0.1325 to 0.4800 g, with an average of 0.2592 g and a variation coefficient of 23.01%. These results indicate an extensive phenotypic variation (PV) in our experimental population. Furthermore, according to the kurtosis and skewness values, all traits showed skewed distribution, especially GR (**Figures 1A, C, E**; **Table 1**). We further analyzed the SV-related values in *indica* and *japonica* subpopulations. It revealed that the GR, SL, and FW of *indica* and *japonica* were 69.94% and 69.42%, 16.4 cm and 14.0 cm, 0.2722 g and 0.2391 g, respectively. Except for GR, a significant difference was observed for SL and FW between *indica* and *japonica* (**Figures 1B, D, F**). In sum, the GR, SL, and FW traits were suitable for subsequent GWAS mapping.

**Table 1:** The SV-related traits in 346 rice accessions.

Phenotype	Mean	Range	Coefficient of variation (%)	Kurtosis	Skewness
GR	69.74%	6.00-100.00%	28.69	0.22	-0.91
SL	15.42 cm	8.40-26.20 cm	22.65	0.34	0.68
FW	0.2592 g	0.1325-0.4800 g	23.01	0.30	0.51

### Population structure of 346 rice accessions

**Figure 2:**
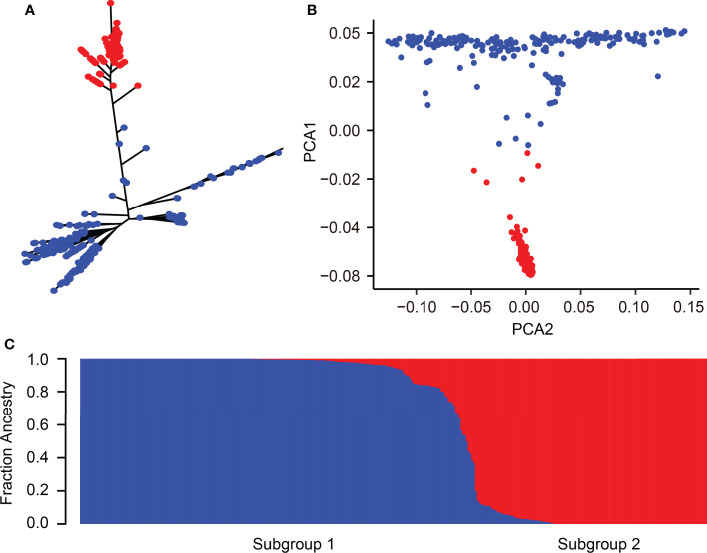
Population structure of 346 rice accessions. **(A)** Phylogenetic tree of 346 rice accessions. **(B)** PCA of 346 rice accessions. The first and second principal components are represented by PCA1 and PCA2, respectively. **(C)** Cluster analysis of 346 rice accessions. Blue and red colors correspond to *indica* and *japonica* accessions, respectively.

The 346 rice accessions were classified into two subgroups based on the phylogenetic tree, roughly corresponding to the classification of *indica* and *japonica* (**Figure 2A**). Similarly, PCA and the population structure base on the possible clusters (k) method also showed that these rice accessions could be divided into two subgroups as the phylogenetic tree classification (**Figures 2B, C**). Taken together, the category of population structure has a similar result compared to a previous study (Shi et al., 2021; Li et al., 2022).

### Mining loci associated with SV by GWAS

**Figure 3:**
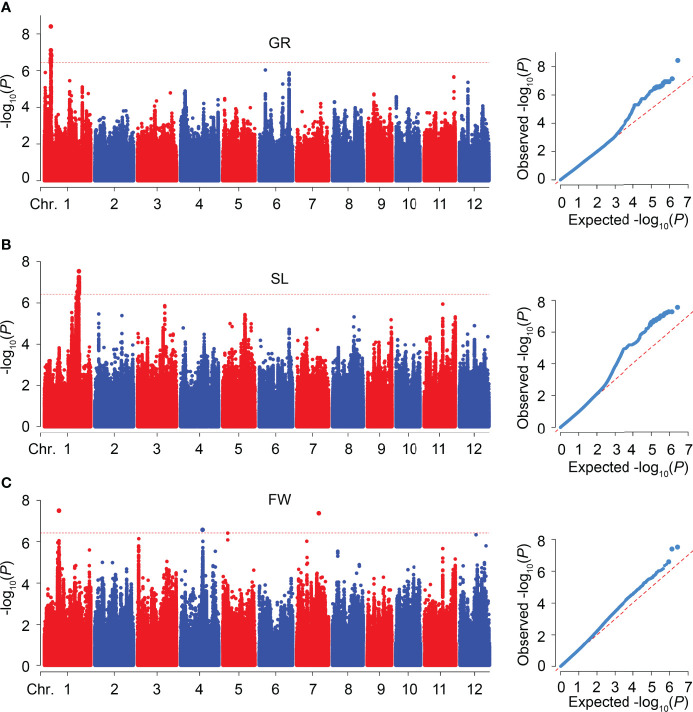
GWAS for SV-related traits in 346 rice accessions. **(A-C)** Manhattan and quantile-quantile (Q-Q) plots of GWAS for GR **(A)**, SL **(B)**, and FW **(C)**, respectively.

To explore the novel loci related to SV, we performed GWAS on GR, SL, and FW in 346 rice accessions, respectively. The Manhattan plots of GWAS showed 18, 30, and 3 significant SNPs were associated with GR, SL, and FW, respectively, and assigned as six QTLs (*qGR1-1*, *qSL1-1*, *qSL1-2*, *qFW1-1*, *qFW4-1*, and *qFW7-1*) according to their physical position in the genome (**Figure 3**; **Table 2** and **Supplementary Table 2**). For GR and SL, 18, 2, and 28 significant SNPs were detected in *qGR1-1*, *qSL1-1*, and *qSL1-2* on chromosome 1, contributing 7.76 ~ 10.53%, 7.92%, and 7.78 ~ 9.33% of the phenotypic variation explanation (PVE), respectively (**Table 2**, **Supplementary Table 2**). Whereas FW, *qFW1-1*, *qFW4-1*, and *qFW7-1* located on chromosomes 1, 4, and 7 respectively, all contain only one associated SNP that explains 7.98 ~ 9.28% of the PVE (**Table 2**, **Supplementary Table 2**).

**Table 2:** Six GWAS regions associated with SV.

QTL	Chr.	Region (nt)	Significant SNPs	Lead SNP		Co-location loci	References
				Position (nt)	-log10 (*P*)		
*qGR1-1*	1	5,689,847-6,629,529	18	5,990,942	8.41	*QAlGC1.2*	Janhan et al., 2021
*qSL1-1*	1	30,535,752-30,935,754	2	30,735,752	6.53		
*qSL1-2*	1	31,306,390-32,339,392	28	32,114,784	7.54	*qASRI1c*	Islam et al., 2022
*qFW1-1*	1	13,379,050-13,779,050	1	13,579,050	7.50	*qSWW1b*	Zhang et al., 2017
*qFW4-1*	4	20,256,553-20,656,553	1	20,456,553	6.58		
*qFW7-1*	7	20,402,053-20,802,053	1	20,602,053	7.38		

### Validation of GWAS-associated loci using CSSLs

**Figure 4:**
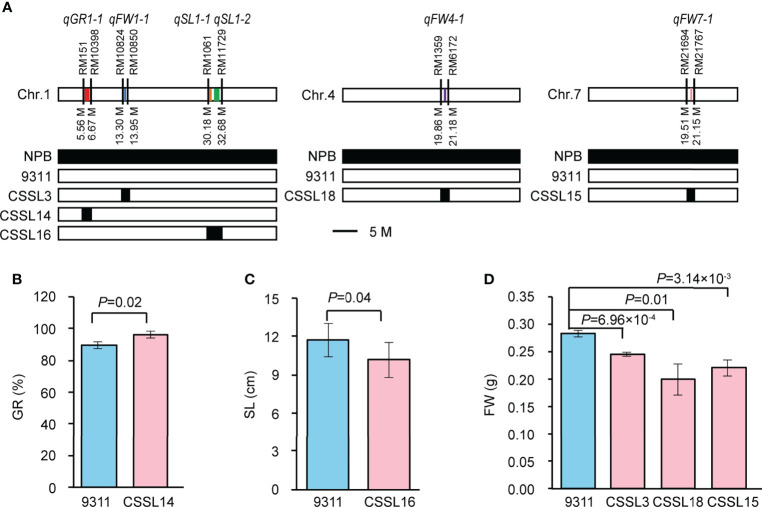
Validation of six GWAS-associated QTLs using CSSLs. **(A)** The genotype diagrams of CSSLs. *qGR1-1*, *qSL1-1*, *qSL1-2*, *qFW1-1*, *qFW4-1*, *qFW7-1* are represented by red, orange, green, blue, purple, and yellow lines, respectively. Line width indicates the size of QTL interval. **(B)** Comparison of GR between CSSL14 and 9311. Data represent mean ± SD (n=3). **(C)** Comparison of SL between CSSL16 and 9311. Data represent mean ± SD (n=10). **(D)** Comparison of FW among CSSL3, CSSL18, CSSL15, and 9311. Data represent mean ± SD (n=3). n indicates the number of replicates. The p-value is obtained from the T-test.

To verify the GWAS-associated QTLs, we screened five CSSLs from the CSSL library with 9311 as recurrent parent and NPB as donor parent. We named them CSSL14 (containing *qGR1-1*), CSSL16 (containing *qSL1-1* and *qSL1-2*), CSSL3 (containing *qFW1-1*), CSSL18 (containing *qFW4-1*), and CSSL15 (containing *qFW7-1*), respectively (**Figure 4A**). A comparison of SV-related traits between 9311 and corresponding CSSLs revealed significant differences in GR, SL, and FW, respectively. In detail, the GR of CSSL14 increased by 7.34% compared with that of 9311 (**Figure 4B**), while the SL of CSSL16 and FW of CSSL3, CSSL18, and CSSL15 decreased by 12.86%, 13.27%, 29.50%, and 22.08% compared with that of 9311, respectively (**Figures 4C, D**). These data suggest that the above GWAS results are reliable and imply that the NPB allele is the dominant allele of *qGR1-1*, whereas the allele from 9311 is the superior allele for *qSL1-1*, *qSL1-2*, *qFW1-1*, *qFW4-1*, and *qFW7-1*.

### Identification of SV-related candidate genes in GWAS-associated loci

To search for the candidate genes in six QTLs, we excavated all 540 annotated genes, including 135, 60, 169, 59, 58, and 59 genes in *qGR1-1*, *qSL1-1*, *qSL1-2*, *qFW1-1*, *qFW4-1*, and *qFW7-1*, respectively (**Supplementary Table 3**). We used three strategies to narrow down the candidate genes: Firstly, 76 genes not expressed in seeds (for GR) or seeds and leaves (for SL and FW) were deleted based on the expression profile in Rice Expression Database (http://expression.ic4r.org/) (**Supplementary Table 3**). Secondly, 65 genes encoding retrotransposon or transposon protein were excluded based on their function annotations (**Supplementary Table 3**). Meanwhile, 28 genes considered highly related to the SV phenotype were screened from the remaining genes (**Supplementary Table 3**). The common feature of these 28 genes is that they and their homologs are at least associated with SV-related traits or involved in hormone metabolism or signaling pathways such as ABA and GA. Lastly, haplotype analysis was carried out using the SNPs in 346 accessions, and 21 candidate genes associated with significant SV-related phenotypic differences between their top two or three haplotypes were screened (**Supplementary Tables 1**, **3**). Their distances from the nearest significant SNPs are shown in **Supplementary Table 4**. However, it is important to note that the phenotypic differences between haplotypes of some candidate genes may result from the phenotypic differences between *indica* and *japonica* subpopulations, since SL and FW differences exist in the two subspecies.

**Figure 5:**
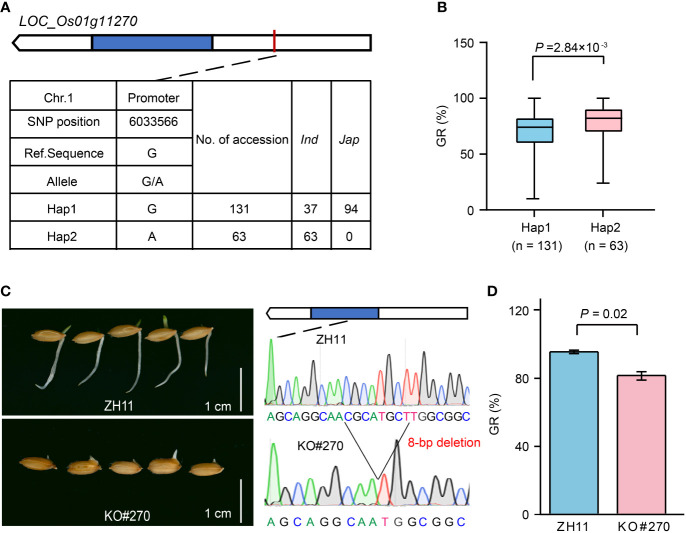
Effect of *LOC_Os01g11270* on rice seed germination. **(A)** The top two haplotypes of *LOC_Os01g11270* in 346 rice accessions. The blue box represents the exon, the white box indicates 2-kb upstream region of the start codon or 1-kb downstream region of the stop codon, and the black line represents the intron. **(B)** Comparison of GR between accessions containing Hap1 and Hap2. **(C)** Comparison of seed germination status after imbibition for five days between *LOC_Os01g11270* gene-edited mutant (KO#270) and its wild-type ZH11. **(D)** Comparison of GR between KO#270 and ZH11. Data represent mean ± SD from three replicates. The p-value is obtained from the T-test.

Among them, *LOC_Os01g11270* located in *qGR1-1* was annotated as a cytochrome P450 (CYP450) (**Supplementary Table 3**), and its homologs played a crucial role in hormone metabolisms, such as gibberellin (GA) (Zhu et al., 2005; Wu et al., 2014) and abscisic acid (ABA) (Zhu et al., 2009; Zhang et al., 2020). GA and ABA are two antagonistic phytohormones with positive and negative regulatory effects on seed germination and seedling establishment, respectively. Therefore, *LOC_Os01g11270* is a strong candidate associated with GR. Haplotype analysis revealed that *LOC_Os01g11270* has two major haplotypes according to an SNP located in its promoter (**Figure 5A**). Haplotype 1 (Hap1, reference sequence) was mainly found in *japonica* accessions, while Hap2 only occurred in *indica* accessions (**Figure 5A**; **Supplementary Table 1**). Comparatively, Hap1 had a lower GR than Hap2 (**Figure 5B**). To further validate the role of *LOC_Os01g11270* in rice seed germination, we compared the GR of the *LOC_Os01g11270* gene-edited mutant (KO#270) and its wild-type Zhonghua 11 (ZH11). In KO#270, an 8-bp deletion at base 799 to 806 in the *LOC_Os01g11270* CDS caused a frameshift mutation (**Figure 5C**). The statistical result showed that the GR of KO#270 decreased by 15% compared to that of ZH11 (**Figures 5C, D**), suggesting a positive regulation effect of *LOC_Os01g11270*.

**Figure 6:**
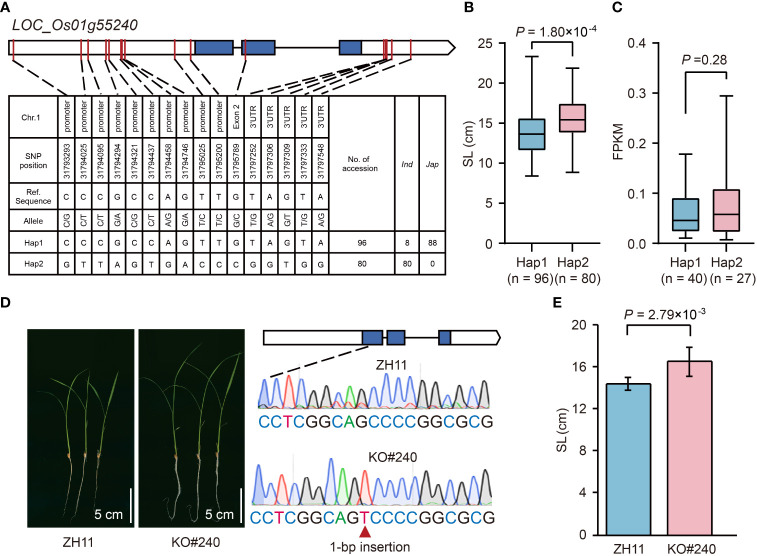
Effects of *LOC_Os01g55240* on rice seedling growth. **(A)** The top two haplotypes of *LOC_Os01g55240* in 346 rice accessions. The blue box represents the exon, the white box indicates 2-kb upstream region of the start codon or 1-kb downstream region of the stop codon, and the black line represents the intron. **(B)** Comparison of SL between accessions with Hap1 and Hap2. **(C)** Comparison of gene expression in leaves between accessions with Hap1 and Hap2. The mean FPKM value of three replicates is represented as the gene expression value for each accession. **(D)** Comparison of seedling growth status after imbibition for ten days between *LOC_Os01g55240* gene-edited mutant (KO#240) and its wild-type ZH11. **(E)** Comparison of SL between KO#240 and ZH11. Data represent mean ± SD from ten replicate. The p-value is obtained from the T-test.

*LOC_Os01g55240* in *qSL1-2* was identified as a GA inactivation gene (**Supplementary Table 3**), encoding a gibberellin 2-beta-dioxygenase (OsGA2ox3) (Tong et al., 2014), and thus may control seedling growth. Haplotype analysis of *OsGA2ox3* revealed two major haplotypes, Hap1 (reference sequence) and Hap2, that existed in 346 rice accessions (**Figure 6A**). The Hap1 associated with short SL was mainly found in *japonica* accessions, whereas Hap2 related to long SL only occurred in *indica* accessions (**Figures 6A, B**). To know whether the differential gene expression levels cause the SL variation in different haplotypes, we compared the transcripts of *OsGA2ox3* between Hap1 and Hap2 accessions using the leaf tissues (the uppermost complete leaves from four 3-week-old plants) RNA-seq dataset from our laboratory. The result showed that there was no significant difference between the average gene expression levels of Hap1 and Hap2 (**Figure 6C**; **Supplementary Table 1**), suggesting that the SL difference between Hap1 and Hap2 was not caused by their differential gene expression levels. To further validate the effect of *OsGA2ox3* in rice seedling growth, we compared the SL between the *OsGA2ox3* gene-edited mutant (KO#240) and its wild-type ZH11. In KO#240, one bp insertion between base 104 to 105 in the *OsGA2ox3* CDS resulted in a frameshift mutation (**Figure 6D**). It showed that the SL of KO#240 was 15% longer than that of the wild type (**Figures 6D, E**), suggesting a negative regulation effect of *OsGA2ox3*.

## Discussion

Seed vigor is crucial in determining the yield of direct seeding rice. Good SV contains strong and uniform seed germination, rapid seedling growth, and increased stress tolerance. In contrast, apart from without these advantages, the poor SV also leads to weed bloom due to the weak seedling competitiveness, which further reduces rice production (Rajjou et al., 2012). Therefore, deciphering the molecular regulatory mechanisms of SV, and identifying the causative genes underlying SV have been the focus of rice biology. In the past decade, GWAS has been widely used to detect the potential loci or alleles underlying complex agronomic traits in rice. This study used three indicators, GR, SL, and FW, closely related to seed germination and seedling growth, to evaluate SV and perform GWAS. The results revealed a considerable variation and nearly normal distribution of GR, SL, and FW in 346 accessions, respectively (**Figures 1A, C, E**; **Table 1**). In addition, the *indica* accessions may be more suitable for DS because they had larger SL and FW than *japonica* accessions (**Figures 1D, F**). A total of six QTLs (*qGR1-1* for GR, *qSL1-1* and *qSL1-2* for SL, *qFW1-1*, *qFW4-1*, and *qFW7-1* for FW) were identified through GWAS using the whole population (**Figure 3**; **Table 2**). Among them, we found two QTLs co-located with previously reported SV-related QTLs using the traditional mapping method, suggesting that our GWAS results are pretty reliable (**Table 2**). For instance, *qGR1-1* corresponds to *QAlGC1.2*, a QTL for germination capacity under aluminum stress and mapped between markers RM14 and RM243 (Jahan et al., 2021). *qFW1-1* shares the same location with *qSWW1b*, a major QTL for the wet seedling weight (Zhang et al., 2017). In addition, we also found *qSL1-2* co-located with previously reported *qASRI1c*, a QTL for anaerobic salt response index (ASRI) of coleoptile identified by GWAS (Islam et al., 2022). However, other GWAS signals do not overlap with the previous GWAS results for SV-related traits (Li et al., 2021a; Peng et al., 2022; Yang et al., 2022). Besides the different rice accessions we used, one possible reason for the different results of GWAS is that phenotypic statistics of previous GWAS were mainly performed in Petri dishes and under constant temperature conditions. In contrast, we conducted phenotypic statistics in field conditions. In this study, we further used CSSLs to validate the GWAS-associated loci, and this strategy is relatively rare in GWAS studies.

By scanning the 540 annotated genes in six QTLs, we screened 21 strong candidate genes associated with SV (**Supplementary Table 3**). Based on functional annotations and literature review, these genes can be broadly classified into hormone homeostasis and signal-related, CYP450s, calcium signal-related, transcription factors, and so on. Here, we chose some representative ones for discussion. For example, *LOC_Os01g11150* (*OsGA2ox7*), *LOC_Os01g55240* (*OsGA2ox3*), *LOC_Os07g34240*, and *LOC_Os07g34370* were annotated as gibberellin 2-beta-dioxygenase or putative gibberellin receptor (**Supplementary Table 3**). Therefore, they may influence SV by regulating GA activity or signaling pathway. In fact, *OsGA2ox3* is mainly expressed in seedlings, and the *OsGA2ox3-*activated mutant or overexpression transgenic plant was severely dwarfed and maintained a vegetative growth (Lo et al., 2008; Takehara et al., 2020; Hsieh et al., 2021). In contrast, *OsGA2ox3* CRISPR/Cas9 knockout mutant showed a taller plant height due to elongated internodes and leaves (Takehara et al., 2020; Hsieh et al., 2021). These results suggest that *OsGA2ox3* is a negative regulator of plant height. Similarly, we also confirmed that *OsGA2ox3* down-regulated SL using *OsGA2ox3* CRISPR/Cas9 knockout mutant (**Figures 6D, E**). Furthermore, we found that the Hap2 in *indica* accessions was the dominant haplotype compared to Hap1 in *japonica* accessions (**Figures 6A, B**), implicating a great potential for improving SV-related traits in *japonica* accessions by *OsGA2ox3*. Compared with *OsGA2ox3*, *OsGA2ox7* in *qGR1-1* is an attenuated functional gene since its overexpression transgenic plants exhibited a semi-dwarf phenotype, whereas no phenotypic change on plant height was observed in *osga2ox7* knockout mutant (Hsieh et al., 2021). Here, we found apparent differences in GR and SL between the two main haplotypes of *OsGA2ox7* (**Supplementary Figures 1A, B**). Similarly, the SL and FW of the two main haplotypes of *LOC_Os07g34240* and *LOC_Os07g34370* in *qFW7-1* also differ significantly (**Supplementary Figures 1C–F**). These results suggest that GA is a vital hormone for regulating SV, and there is a great potential to improve SV by targeting genes related to the GA pathway.

Auxin is a universal coordinator for plant growth and development and stress responses. It is well known to promote coleoptile elongation and seedling growth (Gallei et al., 2020). Of 21 strong candidates, *LOC_Os01g54990* (*OsARF3*), *LOC_Os01g10970*, and *LOC_Os01g55940* (*OsGH3.2*) were regarded as auxin response factor (ARF) or indole-3-acetic acid (IAA)-amido synthetase (conversion of active IAA to an inactive form) (**Supplementary Table 3**), and thus may control SV through modulating auxin homeostasis or signaling pathway. Actually, *OsGH3.2* overexpression not only resulted in remarkable morphological changes in transgenic lines, such as dwarfism, shortened leaf length, and small panicles and internodes, but also showed increased cold stress tolerance and drought hypersensitivity through the modulation of IAA and ABA homeostasis. In contrast, no significant change in these phenotypes was detected in *OsGH3.2*-suppressed plants (Du et al., 2012). Recently, OsGH3.2 was demonstrated as a negative regulator of seed longevity by regulating the accumulation of ABA (Yuan et al., 2021). In this study, we observed significant differences in SV-related traits between the two main haplotypes of *OsGH3.2* and *OsARF3* in *qSL1-2* and *LOC_Os01g10970* in *qGR1-1* (**Supplementary Figures 2A–C**). These data suggest that auxin acts as an essential factor in modulating SV.

*LOC_Os01g11270*, *LOC_Os01g11280*, and *LOC_Os01g11340* in *qGR1-1* were predicted as CYP450s (**Supplementary Table 3**), which are the biggest enzymatic protein family involved in NADPH- and oxygen-dependent hydroxylation reactions in plants (Pandian et al., 2020). As versatile catalysts, CYP450s play vital roles in the biosynthesis of primary and secondary metabolites, antioxidants, and phytohormones, thereby regulating plants’ growth and development, and protecting them from stresses (Xu et al., 2015; Pandian et al., 2020). *CYP714D1*, *CYP714B1*, and *CYP714B2* act as the GA deactivating enzymes in rice, and their mutants and overexpressing transgenic plants showed tall and dwarfed phenotypes, respectively (Zhu et al., 2006; Magome et al., 2013). *CYP90D2* encodes a brassinosteroid (BR) biosynthetic enzyme whose mutants were dwarfed (Hong et al., 2003; Fang et al., 2016). *CYP96B4* modulates rice growth and stress tolerance by finetuning the balance of GA and ABA. *cyp96b4* mutant showed pleiotropic abnormal phenotypes, including dwarfism, delayed germination and early growth, and enhanced tolerance to drought (Tamiru et al., 2015). In addition, *CYP71D8L* is a crucial regulator for GA and cytokinin (CK) homeostasis, thereby controlling multiple agronomic traits and stress responses (Zhou et al., 2020). These results imply that *LOC_Os01g11270*, *LOC_Os01g11280*, and *LOC_Os01g11340* could influence SV by mediating phytohormones’ homeostasis. Here, significant variations in GR between the two main haplotypes of these three genes were detected (**Figures 5A, B**, **Supplementary Figures 3A, B**), which support the above speculation. And the effect of *LOC_Os01g11270* on GR was further confirmed using its CRISPR/Cas9 knockout mutant (**Figures 5C, D**). In addition, the Hap2 with a higher GR may be an elite allele available for SV improvement.

Ca^2+^ acts as an essential nutrient and a universal second messenger for plants’ growth and development in normal and stressful conditions. The Ca^2+^ signals are decoded through the interactions between a unique set of Ca^2+^ sensors named calcineurin B-like proteins (CBLs) and the major downstream signaling components called CBL-interacting protein kinases (CIPKs). These CBLs-CIPKs interactions define the complexity of the Ca^2+^ signaling networks for the perception and transduction of stress signals under various environmental stresses (Tang et al., 2020). In rice, *OsCIPK3*, *OsCIPK9*, and *OsCIPK23* are involved in cold, salinity, and drought stress responses, respectively (Xiang et al., 2007; Yang et al., 2008; Shabala et al., 2021). *OsCIPK31* functions in seed germination and seedling growth under abiotic stress conditions (Piao et al., 2010). *OsCIPK15* is related to rice germination and subflooding tolerance under oxygen deprivation (Lee et al., 2009; Kudahettige et al., 2011). Of 21 strong candidates, two *CIPK* genes, *LOC_Os01g55450* (*OsCIPK12*) in *qSL1-2* and *LOC Os01g10870* (*OsCIPK13*) in *qGR1-1* were observed (**Supplementary Table 3**). We found apparent variations in SL and GR between the two main haplotypes of *OsCIPK12* and *OsCIPK13*, respectively (**Supplementary Figures 4A, C**). In addition, the longer SL in Hap2 of *OsCIPK12* corresponds to the higher gene expression level, indicating that *OsCIPK12* is a positive regulator of SL (**Supplementary Figure 4B**). Our results, combined with the overexpression of *OsCIPK12* resulted in increased drought tolerance in seedlings (Xiang et al., 2007), suggesting that *OsCIPK12* is a promising SV improvement gene.

As critical regulatory elements of gene expression, we identified three transcription factors (TFs) in *qSL1-1* and *qSL1-2*, including one bZIP family TF (*LOC_Os01g55150*) and two OVATE family TFs (*LOC_Os01g53160* and *LOC_Os01g54570*) (**Supplementary Table 3**). The bZIP family proteins have been shown to regulate a set of plant growth and development processes, such as photomorphogenesis, floral induction and development, and seed maturation and germination. They also involve stress responses (Nijhawan et al., 2008). Here, we observed that the Hap2 of *LOC_Os01g55150* had longer SL, heavier FW, and higher gene expression than those of Hap1 (reference sequence) (**Supplementary Figures 5A-C**), implicating that *LOC_Os01g55150* acts as a positive regulator. Compared with the bZIP family, the OVATE family with a conserved DUF623 domain is relatively small and poorly understood. In rice, several OVATE family proteins (OsOFPs) have been cloned and shown to regulate multiple aspects of plant growth and development, including seedling growth, plant architecture, leaf morphology, grain shape, and abiotic stresses (Schmitz et al., 2015; Yang et al., 2016; Ma et al., 2017; Xiao et al., 2017; Yang et al., 2018; Xiao et al., 2020). Among them, *LOC_Os01g53160*, also named *OsOFP3*, acts as a repressor of both BR biosynthesis and signal and incorporates into a TF complex to regulate BR signaling, thereby controlling the proper development of plants. In this study, we found apparent variations in SL and FW between the top two haplotypes of *LOC_Os01g53160* and *LOC_Os01g54570* (**Supplementary Figures 5D–G**). Furthermore, the larger values of SL and FW in Hap2 of *LOC_Os01g54570* were associated with its higher gene expression level (**Supplementary Figure 5H**), implicating a positive role of *LOC_Os01g54570* on SV.

In sum, we detected six QTLs for GR, SL, and FW traits by GWAS within 346 rice accessions, and screened 21 strong candidate genes with differential SV-related features between their top two haplotypes. Two genes, *LOC_Os01g11270* and *LOC_Os01g55240*, were further validated. The identification of those candidate genes and their elite haplotypes provides a promising source for molecular breeding for high vigor seeds in rice.

## Data availability statement

The original SNP data presented in the study are publicly available. This data can be found here: https://github.com/LipingDai/GWAS-for-rice-vigor/. The raw sequencing data presented in this article are not readily available for ownership reasons. Requests to access the datasets should be directed to the corresponding authors.

## Author contributions

DZ, QQ, and QL designed and supervised the study. LD and XL performed the experiments. LD conducted bioinformatics analysis and interpretation of the data. LS, LG, GZ, ZG, LZ, JH, DR, QZ, DZ, QQ, and QL participated in rice material collection and provided resequencing resources. LD and QL drafted the manuscript. DZ revised the manuscript. LD and QL responded to review comments. All authors contributed to the article and approved the submitted version.

## Funding

This work was financially supported by the Hainan Yazhou Bay Seed Laboratory Project (B21HJ0220), the National Natural Science Foundation of China (32101755), the China Postdoctoral Science Foundation (2020M680778), and the Key Research and Development Program of Zhejiang Province (2021C02056).

## Acknowledgments

We thank Biogle Genome Editing Centre (BGEC) for providing the rice gene editing seeds.

## Conflict of interest

The authors declare that the research was conducted in the absence of any commercial or financial relationships that could be construed as a potential conflict of interest.

## Publisher’s note

All claims expressed in this article are solely those of the authors and do not necessarily represent those of their affiliated organizations, or those of the publisher, the editors and the reviewers. Any product that may be evaluated in this article, or claim that may be made by its manufacturer, is not guaranteed or endorsed by the publisher.
