# Supplementary material for: Genome-wide association study reveals novel QTLs and candidate genes for seed vigor in rice
Source: Front Plant Sci. 2022 Oct 26;13:1005203. doi: 10.3389/fpls.2022.1005203 (PMC9645239; doi:10.3389/fpls.2022.1005203)
Supplement: Supplementary file 2 [file Presentation_1.pdf]

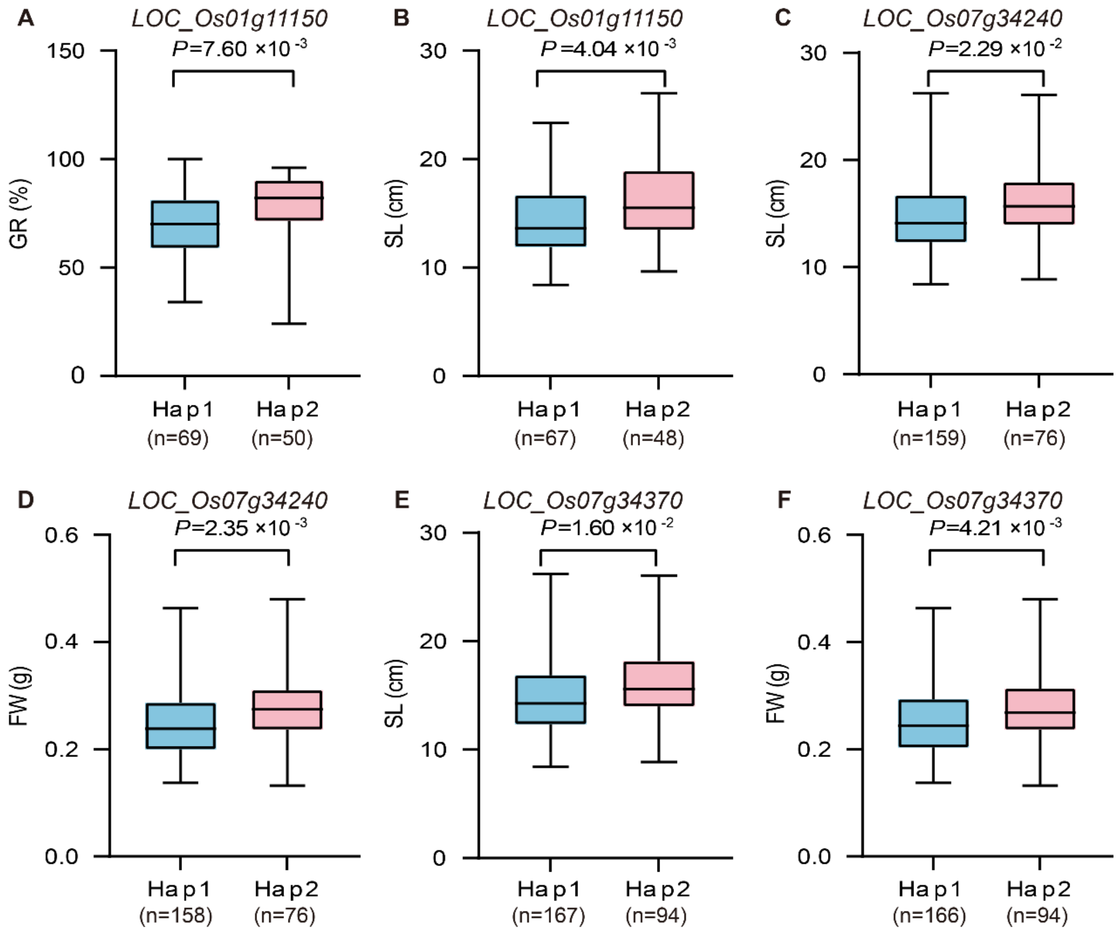

Supplementary Figure 1 Effects of the *LOC_Os01g11150*, *LOC_Os07g34240,* and *LOC_Os07g34370* on rice SV-related traits

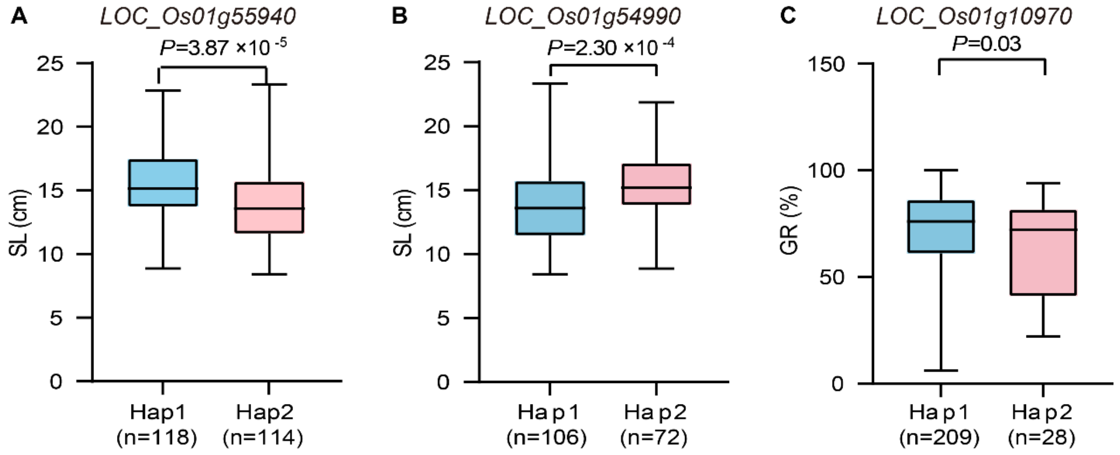

Supplementary Figure 2 Effects of the *LOC_Os01g55940*, *LOC_Os01g54990,* and *LOC_Os01g10970* on rice SV-related traits

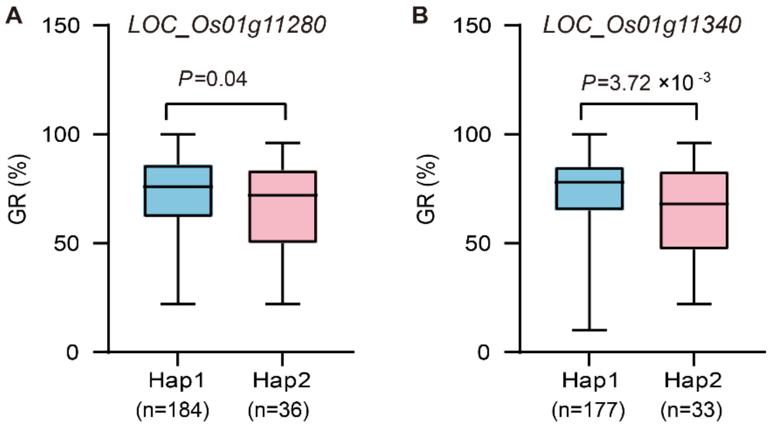

Supplementary Figure 3 Effects of the *LOC_Os01g11280* and *LOC_Os01g11340* on rice SV-related traits

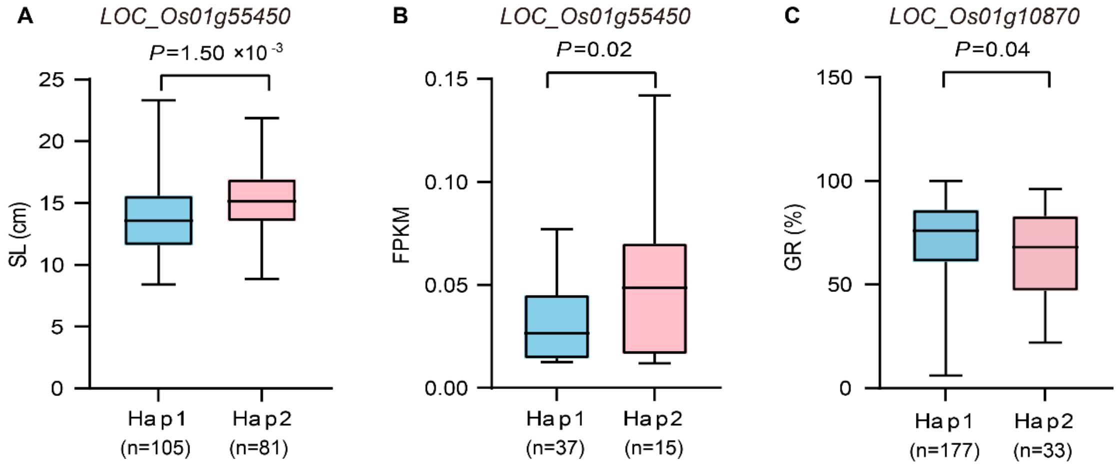

Supplementary Figure 4 Effects of the *LOC_Os01g55450* and *LOC_Os01g10870* on rice SV-related traits

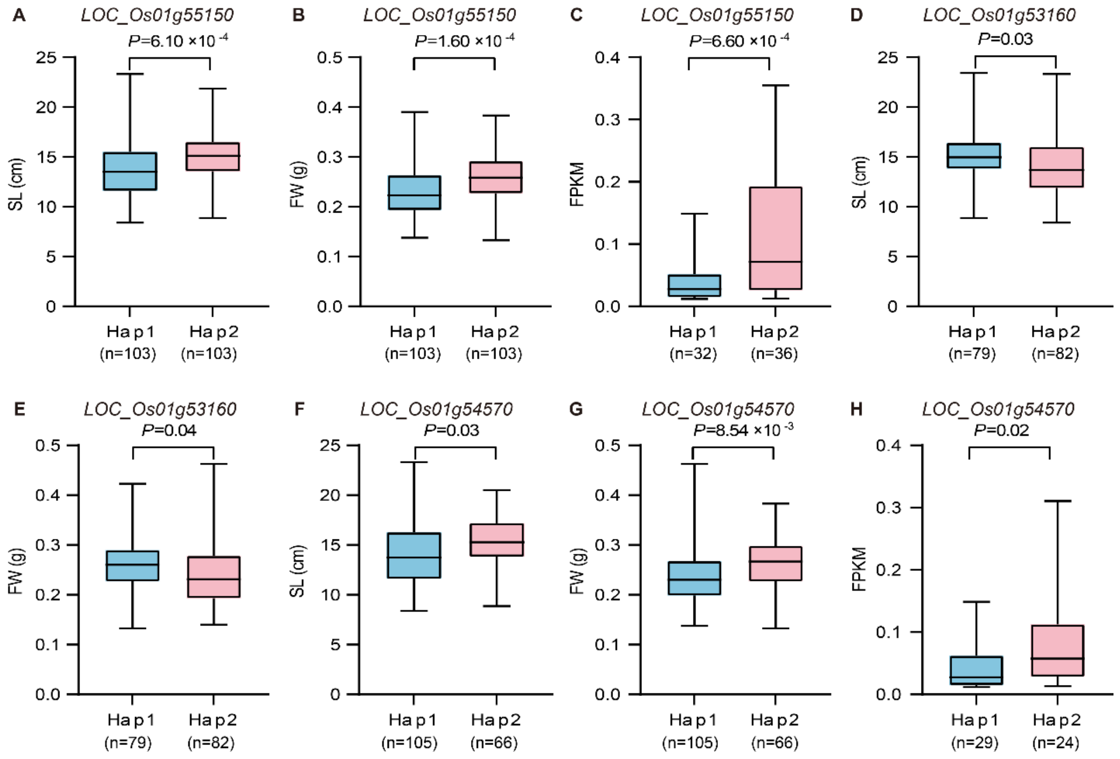

Supplementary Figure 5 Effects of the *LOC_Os01g55150*, *LOC_Os01g53160,* and *LOC_Os01g54570* on rice SV-related traits
